# Feasibility and effect of an immersive virtual reality-based platform for cognitive training in real-life scenarios in patients with mood - or psychotic disorders: A randomized, controlled proof-of-concept study

**DOI:** 10.1016/j.nsa.2023.101120

**Published:** 2023-02-04

**Authors:** Andreas E. Jespersen, Isabella S. Røen, Anders Lumbye, Merete Nordentoft, Louise B. Glenthøj, Kamilla W. Miskowiak

**Affiliations:** a Neurocognition and Emotion in Affective Disorders (NEAD) Centre, Psychiatric Centre Copenhagen, Copenhagen University Hospital, Rigshospitalet Blegdamsvej 9, DK-2100, Copenhagen, Denmark; b Department of Psychology, University of Copenhagen, Øster Farimagsgade 2A, DK-1353, Copenhagen, Denmark; c Wide Angle Media, Copenhagen, Denmark; d Research Center for Mental Health (CORE), Copenhagen, Denmark; e Faculty of Health and Medical Sciences, University of Copenhagen, Copenhagen, Denmark

**Keywords:** Virtual reality, Cognition, Cognitive remediation, Depression, Bipolar disorder, Psychosis

## Abstract

**Objectives:**

Cognitive impairment is common across mood disorders (MD) and psychosis-spectrum disorders (PSD) but there is a lack of real-life pro-cognitive training programmes. Fully immersive virtual reality (VR) has the potential to ensure motivating, engaging cognition training directly relevant to patients’ daily lives. This randomized, controlled proof-of-concept study investigated the feasibility and cognitive benefits of short-term VR-assisted training.

**Methods:**

Forty patients with MD or PSD were randomized to one week of VR-assisted training (n ​= ​20) or treatment as usual (TAU; n ​= ​20). They were assessed at baseline and after one week with a VR cognition test, neuropsychological tests, and questionnaires regarding user experience. Patients in the training group underwent two VR training sessions in a kitchen environment that involved solving tasks related to planning and cooking a meal using various cognitive strategies. They also completed two home assignments during which they applied the acquired strategies.

**Results:**

The completion rate was 100%. Patients reported high enjoyment and moderate-to-high presence in the VR environment and minimal motion sickness. VR training improved the global VR-based cognitive composite score with a large effect size compared with TAU (*F*(1, 38) ​= ​11,29, *p* ​= ​.002, *η*^*2*^ ​= ​0.23). Posthoc assessments of VR subtests showed that this improvement was driven primarily by a large effect on psychomotor speed (*F*(1, 38) ​= ​22.78, *p* ​< ​.001, *η*^*2*^ ​= ​0.39), but no effects were observed on other VR subtests or on traditional neuropsychological tests.

**Conclusion:**

VR-assisted cognition training showed high feasibility and improved aspects of cognition after only one week. We therefore plan a larger trial to investigate the cognitive benefits of four-weeks VR-assisted cognition training.

## 1 Introduction

Cognitive impairment is a core feature of mood disorders (MD), and psychosis spectrum disorders (PSD) (Bora and Pantelis, 2015; Bourne et al., 2013; Rock et al., 2014). The impairment is evident across several cognitive domains, including memory, attention, processing speed, and executive functions and often persists during asymptomatic phases of illness (Bourne et al., 2013; Rock et al., 2014; Bora et al., 2013; Cullen et al., 2016). Cognitive impairment is associated with poorer symptom prognosis, lower quality of life, and more functional disability (Jaeger et al., 2006; Jaeger and Vieta, 2007; Tse et al., 2014). In particular, the reduced work force capacity in these patient groups comprises the largest socio-economic burden of the disorders (Olesen et al., 2012; Chong et al., 2016). Cognitive impairment is therefore an urgent strategic treatment target to improve patients’ functioning and quality life and reduce societal costs (Bowie et al., 2006; Miskowiak et al., 2018).

Cognitive remediation (CR) is a therapeutic intervention that aims to improve functional recovery by targeting cognitive impairment through cognitive training, cognitive rehabilitation, and cognitive stimulation (Tsapekos et al., 2020; Woolf et al., 2022). CR focusing on cognitive training is a particularity promising approach with well-established effect on cognitive outcomes in PSD (Reser et al., 2019; Wykes et al., 2011) and MD (Lewandowski et al., 2017; Strawbridge et al., 2021). Despite overall moderate cognitive benefits, many CR interventions revealed *limited transfer* of the cognitive gains to functional improvements, which call into question the clinical impact of the interventions (Woolf et al., 2022; Lewandowski et al., 2017; Miskowiak et al., 2022a). A key challenge is that CR rarely involves any *direct* training of how to solve cognitive challenges in real-life situations. Interestingly, a meta-analytic study in schizophrenia indicated that transfer may be improved when CR was combined with structured psychosocial rehabilitation (Vita et al., 2021). However, integrating more direct psychosocial or vocational training is costly and it is therefore pertinent for research to focus on developing and integrating more cost-effective techniques to aid transfer effects (Tsapekos et al., 2020; Miskowiak et al., 2022a). Another issue that may hinder treatment efficacy is that it is often difficult to motivate patients sufficiently to complete the assigned training during these interventions (Glenthoj et al., 2017). Participants may lose interest in the training because they do not experience the relatively abstract computer exercises as meaningful for their cognitive challenges in daily life. As such, CR programmes often suffer poor treatment adherence and high attrition rates (Lewandowski et al., 2017; Glenthoj et al., 2020). These challenges highlight a need for more targeted and real-life-like cognition training programs to increase patients’ real-life cognitive abilities and treatment engagement (Miskowiak et al., 2022a; Piskulic et al., 2015).

Virtual reality (VR) provides a training platform that could help accommodate these challenges by enabling more real-life-like and engaging, ‘gamified’ cognition training. In VR, users can interact with a simulation of naturalistic and multimodal cognitive challenges that are similar to real-life situations using a relatively cost-efficient and automated technology (Biocca, 1992; Bohil et al., 2011; Lee and Wong, 2014). VR therefore seems a promising treatment modality for more ecologically valid cognitive training within simulated daily-life scenarios that are more relevant to the everyday life of the patient (Bohil et al., 2011). It seems reasonable to assume, that such increased bridging between cognitive training and daily-life functioning may facilitate greater transfer effects of cognitive gains (Tieri et al., 2018). Another promising aspect of VR is the positive impact on patient motivation. Fully immersive VR, such as Head Mounted Display (HMD), provides an increased experience of being ‘present’ in the virtual environment by effectively shutting out the physical reality (Cummings and Bailenson, 2016). Notably, greater sense of ‘presence’ in immersive VR has been linked to increased engagement in VR users (Makransky and Lilleholt, 2018). In general educational contexts, studies have demonstrated that VR may help aid motivation by boosting entertainment and enjoyment (Makransky et al., 2019, 2020). Similar effects in VR-assisted cognitive remediation programmes could therefore help to increase treatment adherence.

In a recent systematic review, we found initial promising evidence for use of immersive VR-based cognitive training in neuropsychiatric disorders (Jahn et al., 2021). However, no cognitive remediation intervention has yet explored the potential benefits of fully immersive VR for training cognitive functions in MD, and cognitive intervention studies in PSD using VR are still scarce (Jahn et al., 2021). To address this knowledge gap, we developed a fully immersive VR *prototype* training scenario, in which participants train various cognitive functions while preparing a meal in a virtual kitchen environment.

In this randomized controlled proof-of-concept study, we aimed to investigate the feasibility and possible cognitive benefits of this VR prototype training scenario in a sample of patients with MD or PSD who were relatively symptom stable. We hypothesized (i) that the VR training scenario would be feasible, tolerable, and engaging for participants, as reflected by high completion rates and positive user-experience reports and (ii) that one-week of intensive VR-assisted training would lead to cognitive improvements, as assessed with a novel VR test of real-life cognitive functions. For exploratory purposes, we also investigated training-related changes within cognitive subdomains measured with the VR test and a standard neuropsychological test battery.

## 2 Methods

### 2.1 Participants and recruitment

Participants were referred to the study by specialized psychiatrists at the outpatient Copenhagen Affective Disorder Clinic or the outpatient, early intervention clinics for psychotic disorders (OPUS) in the Capital region of Denmark. Participants in the MD group had an ICD-10 diagnosis of either unipolar disorder or bipolar disorder and were in either full or partial remission as reflected by scores ≤14 on the Hamilton Depression Rating Scale 17 items (HRDS-17) (Hamilton, 1960) and on the Young Mania Rating Scale (YMRS) (Young et al., 1978). Participants in the PSD group had an ICD-10 diagnosis of either schizophrenia or schizotypal disorder and were assessed to be relatively symptom stable by a clinician at OPUS upon study inclusion. All participants were between 18 and 60 years of age and were fluent in Danish. Exclusion criteria included current substance use disorder, comorbid neurological disorder, severe somatic illness, dyslexia, a daily use of benzodiazepines >22.5 ​mg oxazepam or >7.5 ​mg diazepam per day and electroconvulsive therapy within the past three months. Data from *N* ​= ​40 healthy controls (HC) from our previous study (Miskowiak et al., 2022b) with no personal or first-degree family history of psychiatric illness were included to standardize cognitive tests scores. Data security approval was obtained from the Danish Data Protection Agency (VD-2018- 468) and an appropriate ethical committee stated that the study required no approval because it involved no invasive procedures. Written informed consent was obtained from all participants after fully explaining the study procedure.

### 2.2 Study procedure

**Fig. 1:**
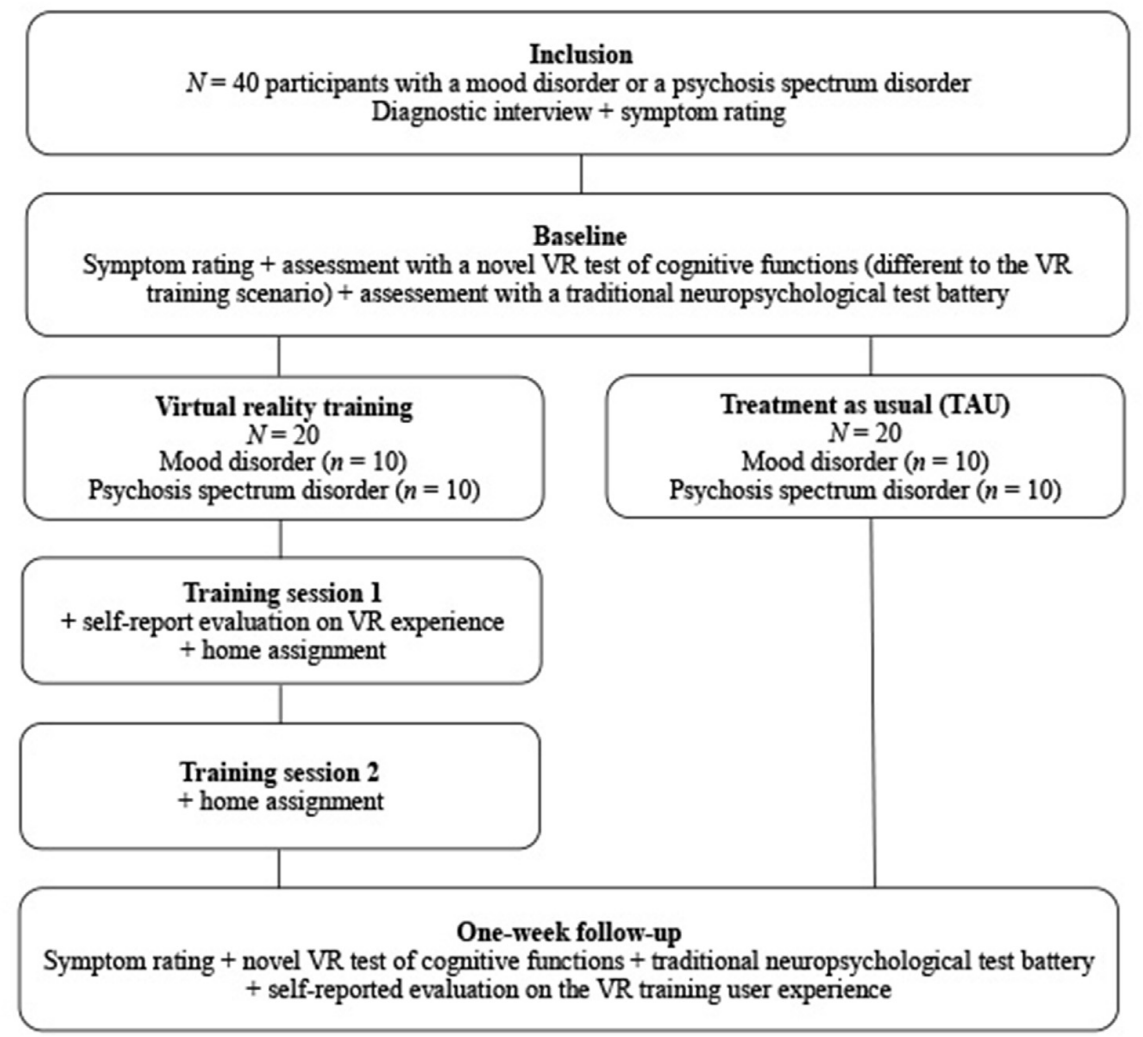
Flow chart of the study design.

The study had a randomized-controlled, parallel group design (See Fig. 1 for flow chart of the study design). Participants were randomized based on a 1:1 inclusion ratio stratified for diagnosis, using a simple pre-fabricated distribution key. Thus, every other patient in each patient group was allocated to short-term VR-assisted training (1 week) or a passive control group receiving treatment as usual (TAU), respectively, in a consecutive manner. Participants in the training group underwent 2 ​× ​90 ​min training sessions with the CAVIR prototype training scenario of real-life cognitive function combined with homework (details below). The training took place at two separate days during the week leading up to the follow-up assessment, respectively. Participants allocated to TAU received no cognitive training or treatment during participation and were simply re-assessed one week (± ​one day) after baseline assessment.

At baseline and follow up, participants with MD underwent mood ratings with the HDRS-17 and YMRS. For participants with PSD, positive symptoms were assessed using the Scale for the Assessment of Positive Symptoms (Andreasen, 1984) and negative symptoms using the Scale for the Assessment of Negative Symptoms (Andreasen, 1989). Feasibility was assessed with questionnaires regarding tolerability and user experience of the VR training program (details below). Cognition was assessed using a novel VR cognition test and a traditional neuropsychological test battery (details below).

### 2.3 Virtual reality assisted cognitive training

**Fig. 2:**
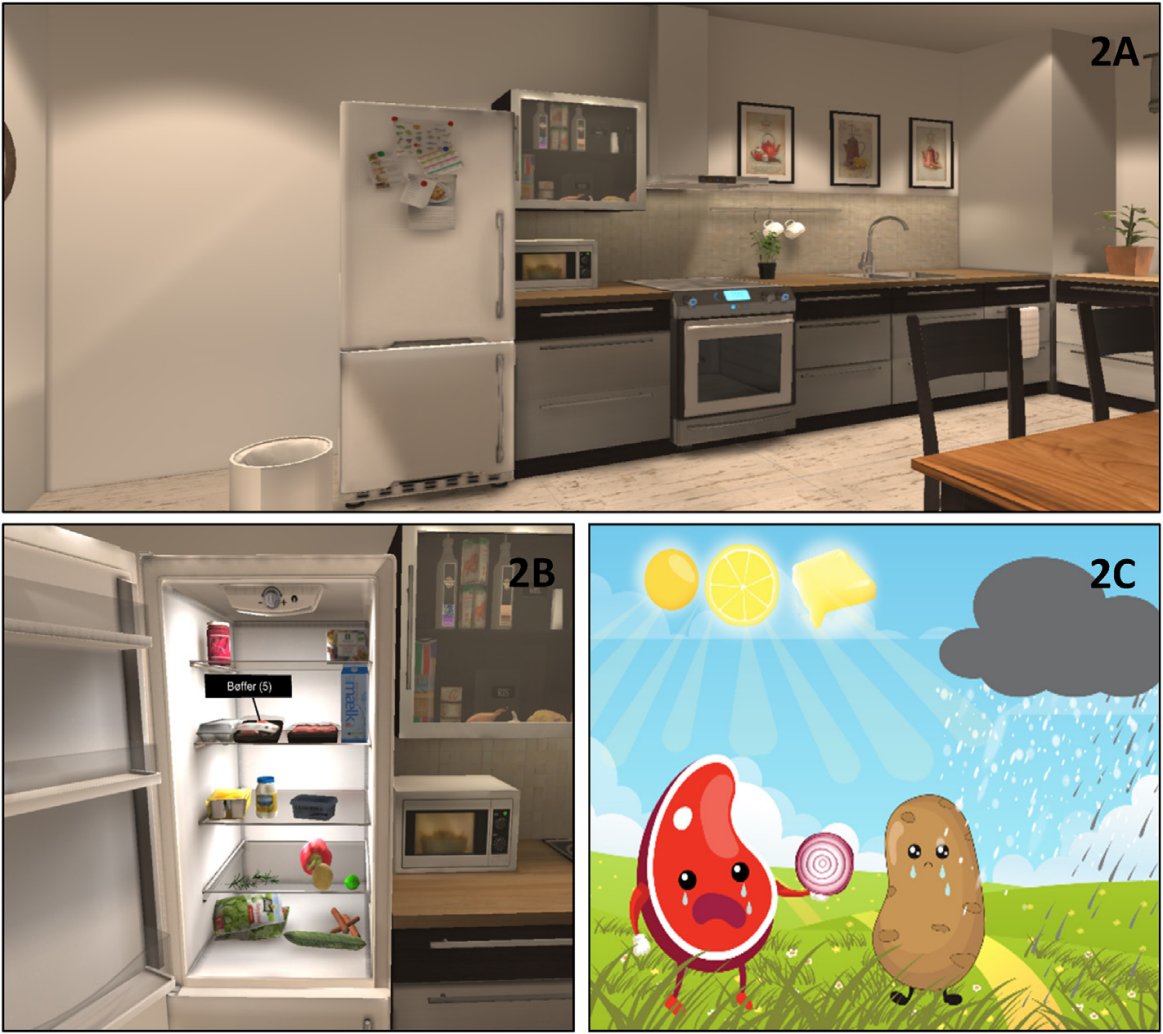
The Cognition Assessment in Virtual Reality (CAVIR) *training* scenario. Note**. 2A:** The immersive 360° VR CAVIR scenario as displayed in an Oculus Go head mounted display. In this setting, participants are tested on - and train - their ability to plan and prepare a meal. **2B**: The VR kitchen scenario during the verbal learning training in which participants must memorize a list of ingredients and find them in the fridge and cupboard. **2C**. Example of a cognitive remediation strategy for training verbal learning and memory through visualizing a story with ingredients.

The intervention comprised two 90 ​min sessions with a trained therapist combined with home assignments. In each session, participants were first introduced to the VR equipment and then trained with the headset on for approximately 45 ​min. The VR training scenario is based in an immersive, interactive kitchen environment administered on a standalone head-mounted Oculus Go 32 ​GB portable headset using a hand-held controller (Fig. 2). In the scenario, participants are instructed through pre-recorded audio instructions to train five different tasks involved in planning and preparing a meal. Participants train verbal learning (memorizing and locating ingredients), executive functions (planning the order of different subtasks), processing speed (placing correct ingredients as quickly as possible in a pot), working memory (memorizing the location of cutlery and flatware throughout the kitchen) and sustained attention (repeatedly monitoring the cooking in response to a specific combination of visual and auditive cues). In addition, the training scenario includes cognitive remediation strategies embedded in the VR-environment that are presented to the participant before each task (Fig. 2C). Examples of strategies include slowing down in the interest of correct performance, visualizing or categorising items to aid encoding and focusing on one stimulus or subtask at a time. After each strategy presentation, participants are instructed to take off their headset to discuss and practice the strategies with the therapist outside the virtual environment before then solving the task in VR. The chosen strategies are based on clinical experience with patients with psychiatric disorders, specifically by conducting cognitive remediation groups focusing on training specific cognitive domains such as verbal learning and executive functioning (Ott et al., 2021). In the last part of each session, participants were instructed on how to complete their home assignment, which involved preparing and cooking a meal at home whilst using the strategies practiced in the sessions. Participants were given a home assignment after each session, thus completing two different meals at home on two separate days during their training.

### 2.4 Measures

#### 2.4.1 Assessment of the experience in the VR training scenario

To assess user-experience and the degree to which participants experienced ‘presence’ in the VR training scenario, participants in the training group completed a shortened version of the Presence Questionnaire (PQ) (Witmer and Singer, 1998; Witmer et al., 2005) after their first training session. They also filled out the VR Simulation Sickness Questionnaire (VRSSQ) (Kim et al., 2018) to assess tolerability. At the one-week follow up assessment, participants subjective feeling of satisfaction and enjoyment with the VR training was assessed using 10-point VAS scales created by our group. Qualitative feedback was subsequently collected from participants who chose to elaborate on their experience with the VR training scenario in a written feedback form. These key insights were translated from Danish to English for the purpose of this article.

#### 2.4.2 Cognitive assessment in virtual reality

Cognition was assessed at baseline and after one week with the Cognition Assessment in Virtual Reality (CAVIR) test which has a duration of 15 ​min and involves five subtasks in a different VR kitchen scenario probing verbal memory, executive functions, processing speed, working memory and sustained attention, respectively (for details see Supplementary materials Table A.1 in Appendix A) (Miskowiak et al., 2022b). The CAVIR test was recently shown to have high validity, sensitivity, and feasibility for cognitive assessment in MD and PSD (Miskowiak et al., 2022b). It can thus be considered an attractive alternative to neuropsychological assessment as it enables insight into patients’ *real-life cognitive functions* across multiple domains (Miskowiak et al., 2022b). For this reason, we defined a global cognitive composite score comprising all five CAVIR subtasks as our primary cognitive outcome in line with methodological recommendations for cognition trials by the International Society for Bipolar Disorder (ISBD) targeting cognition task force (Miskowiak et al., 2017). Importantly, the VR training scenario and CAVIR test differ in their content although they both take place in a VR kitchen scenario. This is to reduce any potential learning effect from repeated exposure to the same VR scenario. Specific differences between the test and training scenarios include the kitchen design and setup (e.g., different interior and placement of items) and test stimuli as different meals are prepared (e.g., different ingredients, and visual/auditive cues).

#### 2.4.3 Neuropsychological assessment

Cognition was also assessed using a traditional neuropsychological test battery comprising the One Touch Stocking of Cambridge (OTS), Spatial Working Memory (SWM) test and Rapid Visual Information Processing (RVP) from CANTAB (Cambridge Cognition Ltd.), Rey Auditory Verbal Learning Test (RAVLT) (Schmidt, 1996), WAIS-III letter-number sequencing (Wechsler, 1997), RBANS Coding and digit span (Randolph et al., 1998), verbal fluency (‘d’ and ‘s’) (Borkowski et al., 1967) and Trail Making A and B (Army Individual Test Battery, 1944). Verbal intelligence was estimated with the Danish Adult Reading Test (DART) (Blair and Spreen, 1989).

### 2.5 Statistical analyses

Group comparisons for demographic data were performed using independent t-tests or Mann-Whitney (normality of data was assessed using Shapiro-Wilk). A χ2 test was applied to investigate potential differences in gender distribution between groups. Adjustments for baseline differences were only performed in case of severe imbalance between groups for variables with effect on cognition.

Scores on the CAVIR test and traditional neuropsychological tests were z-transformed based on the mean and standard deviation of a healthy control group from our previous study (Miskowiak et al., 2022b). For the CAVIR test, five cognitive domains were calculated by averaging the z-transformed scores within each of the five subtasks. A global CAVIR composite score was then calculated by averaging the five cognitive domains. Five cognitive domains were also calculated based on the z-transformed neuropsychological test scores within the respective domains. For further details, see Supplementary Material in Appendix A.

Participants cognitive performance at baseline were compared to the performance of the healthy control group using independent sample t-tests. Effects of the intervention was investigated with repeated-measures ANOVA with group (active, TAU) *x* time (baseline, 1 week) interaction for the CAVIR composite score (primary outcome). The same analyses were also carried out for the five CAVIR domains and traditional neuropsychological domains. Given that these additional subdomain analyses were explorative and only included for hypothesis generating purposes, they were not adjusted for multiple comparisons. Finally, Pearson's correlation analysis was used to investigate the association between cognitive impairment at baseline and cognitive improvement in the intervention group.

All statistical analyses were carried out using IBM SPSS statistics (version 25; IBM Corporation, Armonk, New York). Statistical significance was set to an alpha-level of p ​< ​.05 (two tailed).

## 3 Results

### 3.1 Participant flow and group characteristics

A total of 40 participants were included with 20 participants randomized to VR-assisted training (MD ​= ​10, PSD ​= ​10) and 20 participants randomized to the TAU group (MD ​= ​10, PSD ​= ​10). All participants were assessed at baseline and after one week. Demographic and clinical data is presented in Table 1. Comparisons between the two groups revealed that the two groups were comparable for age, gender, education, premorbid IQ, symptoms of mania (MD only) and positive symptoms (PSD only) (*p*_*s*_ ​≥ ​.45). All participants with MD were in at least partial remission at baseline, but MD participants in the TAU group displayed more subsyndromal symptoms (*t*(18) ​= ​2.23, *p* ​= ​.04) compared to MD participants in the training group. In contrast, participants with PSD in the training group displayed more negative symptoms at baseline assessed with SANS (*t*(18) ​= ​−2.26, *p* ​= ​.04).

**Table 1:** Demographic and clinical characteristics at baseline.

	Group		*p*-value
	VR training	TAU group	
	*(n ​= ​20)*	*(n ​= ​20)*	
**Demographic and clinical variables**			
Diagnoses MD (UD/BD)/PSD (SZ/ST)	10 (3/7)/10 (4/6)	10 (1/9)/10 (3/7)	
Age, mean (SD)	32.0 (11.7)	30.7 (12.2)	.733
Years of education, mean (SD)^a^	13.8 (2.0)	13.5 (2.3)	.641
Est. premorbid intellectual ability, mean (SD)^b^	109.1 (6.6)	110.2 (5.6)	.542
Gender, no women (%)	12 (57.1)	10 (47.6)	.537
HDRS-17 baseline, mean (SD)	3.9 (3.2)	8.2 (5.1)	**.041∗**
YMRS baseline, mean (SD)	1.1 (2.3)	1.8 (3.2)	.467
SAPS Psychosis, mean (SD)	3.7 (3.1)	4.5 (4.1)	.447
SAPS Disorganized, mean (SD)	1.7 (2.0)	1.6 (2.3)	.840
SANS, mean (SD)	3.8 (1.7)	2.3 (1.2)	**.036∗**
No. of participants with cognitive impairments at baseline (score >1 SD below HC mean (%)^c^	6 (30%)	4 (20%)	.465
No. of participant with cognitive impairment at baseline (score >0.5 SD below HC mean (%)^c^	12 (60%)	7 (35%)	.113

Note. MD: mood disorders UD: unipolar disorder, BD: bipolar disorder; PSD: Psychosis spectrum disorders, SZ: schizophrenia, ST: schizotypal disorder, HDRS17: Hamilton Depression Rating Scale 17 items, YMRS: Young Mania Rating Scale, SAPS: Scale for Assessment for Positive Symptoms, SANS: Scale for Assessment for Negative Symptoms, SD: Standard deviation, HC: healthy controls ∗ p ​< ​.05 (two-tailed).

a Missing data for *n ​=* 1 participant in TAU with MD.

b Assessed through the Danish Adult Reading Test (DART). Missing data for *n ​=* 1 participant in TAU with PSD.

c Cognitive impairment is defined as a score either >1 SD or >0.5 SD below the mean of a healthy control group on the CAVIR cognitive composite score (Miskowiak et al., 2017).

### 3.2 Feasibility of the VR-assisted cognitive training

The attendance rate across the two training sessions were 100%. However, only 75% of participants in the training group reported that they had completed *both* homework activities (i.e., prepared two separate meals at home). Reasons for non-completion of the homework included forgetting the assignment or practical difficulties related to cooking at home on the specific days of the assignments.

Results on the self-report measures regarding feasibility and usability are presented in Table 2. In the training group, *n* ​= ​19 participants completed the PQ and VRSSQ after their first training session. On the PQ, participants reported moderate to high perceived presence in the CAVIR training scenario (*M* ​= ​5.2, *SD* ​= ​0.7 of the maximum score of 7.0) and good usability of the headset and controller (*M* ​= ​5.3, *SD* ​= ​1.0 of the maximum score of 7.0). Participants reported almost no ‘simulation sickness’ in the training scenario as suggested by low scores on the VRSSQ (*M* ​= ​0.4; *SD* ​= ​0.3 of the maximum score of 4.0).

**Table 2:** Mean scores for self-report measures assessing the experience in the CAVIR training environment for participants in the VR training group (n=20).

**PQ (score range: 0**–**7)**^a^	
Overall degree of ‘presence’, M±SD	5.2 ​± ​0.7
Natural, M±SD	4.7 ​± ​1.2
Involvement, M±SD	5.3 ​± ​0.7
Interface Quality M±SD	5.3 ​± ​1.0
**VRSSQ (score range: 0**–**4)**^b^	
Overall experience of ‘simulator sickness’, M±SD	0.4 ​± ​0.4
**VAS-scale items**^c^	
1. How satisfied are you overall with the virtual reality training in the kitchen scenario? M±SD	89 ​± ​12%
2. How fun was it to train your cognitive abilities in the virtual reality training kitchen scenario? M±SD	72 ​± ​19%

Note. PQ = Presence Questionnaire (Witmer and Singer, 1998). VRSSQ = Virtual Reality Simulator Sickness Questionnaire (Kim et al., 2018). PQ, Responses for the PQ were rated on a 7-point scale ranging from 1(low degree) to 7 (high degree). Responses for the VRSSQ were rated on a 4-point scale ranging from 0 (none) to 4 (severe). Responses for the two statements regarding satisfaction and engagement were rated on a classic 100-mm VAS scale with the endpoints being “very unsatisfied/very satisfied” and “not fun at all/very fun”, respectively.

a Data missing for 1 participant with MD.

b Data missing for 1 participant with PSD.

c Data missing for 1 participant with MD and 1 participant with PSD.

Participants reported high perceived satisfaction with the training scenario (89% ​± ​12%; Table 2). 13 out of the 20 participants in the training group chose to elaborate on their experience in the written feedback form. Of these 13 participants, 85% noted that they found the training useful in their daily life. As one participant with MD noted: “*The strategy with visualizing a funny story helped me in the supermarket. I often forget to write a shopping list and have to remember what to buy”.* Participants also reported moderate to high enjoyment in the training scenario (72 ​± ​19%; Table 2) and 54% of the participant who completed the written feedback form noted that the training felt entertaining. As one participant with PSD noted: “*It is fun with VR. I learn better when it is fun. The tasks in VR felt more like a game and that helps you relax more”.* For all key insights, see Supplementary materials in Appendix A.

### 3.3 Cognition performance at baseline

Baseline performance on the CAVIR test across the two groups are presented in Table 3 (for figure results, see Supplementary materials Figure A.1 in Appendix A). At baseline, participants displayed significantly lower performance compared to HCs on the CAVIR cognitive composite score in both the VR training group (*t*(58) ​= ​−4.27, *p* ​= ​.001, *g* ​= ​0.74) and TAU group (*t*(58) ​= ​−2.19, *p* ​= ​.03, *g* ​= ​0.33). For the CAVIR subdomains, participants in the VR training group showed significantly lower performance on processing speed *t*(57) ​= ​−4.59, *p* ​< ​.001, *g* ​= ​1.31) and attention (*t*(57) ​= ​−2.60, *p* ​= ​.01, *g* ​= ​0.74), while participants in the TAU group only displayed impairment on processing speed (*t*(57) ​= ​−2.37, *p* ​= ​.03, *g* ​= ​0.67). Regarding the proportion of participants with clinically relevant objective cognitive impairment across the two groups at baseline, six (30%) participants in the active group and four (25%) in the TAU group had a CAVIR global cognitive score >1 SD below the mean of HCs, while 12 (65%) participants in the active group and seven (35%) in the TAU group had a score >0.5 SD below the HC mean. There were no significant difference between the VR training group and TAU group on any of the CAVIR measures at baseline (*p*_*s*_ ​≥ ​.07).

**Table 3:** Cognitive effect of CAVIR training vs. TAU (primary and explorative outcomes).

	Baseline	One-week follow up	Change z-score (Δ)	Treatment effect (time∗group)			Hedge's *g*
	M (SD)	M (SD)		F	*P*-value	ηp2	
Primary outcome							
CAVIR test composite score				11.29	**.002∗**	.229	1.04
Training	−0.7 (0.8)	0.5 (0.6)	1.2				
TAU	−0.3 (0.6)	0.2 (0.5)	0.5				

Exploratory outcomes							
CAVIR test domains							
Verbal learning and memory^a^				.03	.861	.001	0.05
Training	−0.3 (1.2)	0.0 (0.8)	0.3				
TAU	−0.3 (1.0)	0.0 (0.8)	0.3				
Executive functions				1.61	.212	.041	0.39
Training	−0.2 (1.1)	0.5 (0.3)	0.7				
TAU	−0.1 (1.1)	0.3 (0.6)	0.4				
Psychomotor speed^b^				22.78	**< .001∗∗**	.394	1.54
Training	−1.5 (1.4)	1.5 (1.5)	3.0				
TAU	−0.7 (1.1)	0.2 (1.3)	0.9				
Working memory				.07	.788	.002	0.08
Training	−0.7 (1.6)	0.5 (0.7)	1.2				
TAU	−0.5 (1.4)	0.5 (0.5)	1.0				
Attention				.45	.509	.012	0.22
Training	−0.8 (1.2)	−0.2 (1.3)	0.6				
TAU	−0.2 (0.8)	0.1 (0.8)	0.3				
Neuropsychological test domains							
Verbal learning and memory				3.08	.088	.075	0.54
Training	−0.6 (0.9)	−0.2 (0.6)	0.4				
TAU	−0.5 (1.1)	−0.4 (1.3)	0.1				
Executive functions				.01	.981	.000	0.00
Training	−0.8 (0.6)	−0.7 (0.7)	0.1				
TAU	−0,4 (1.0)	−0.3 (0.9)	0.1				
Psychomotor speed				3.16	.083	.077	0.55
Training	−1.2 (0.7)	−1.1 (0.9)	0.1				
TAU	−0.8 (1.2)	−0.3 (0.9)	0.5				
Working memory				0.82	.370	.021	0.28
Training	−0.4 (0.8)	−0.3 (0.8)	0.1				
TAU	−0.4 (0.8)	−0.2 (0.8)	0.2				
Attention				0.91	.347	.023	0.30
Training	−0.1 (0.4)	−0.1 (0.5)	0.0				
TAU	−0.3 (0.6)	0.1 (0.6)	0.2				

Note. ∗p ​< ​.05 (two-tailed). ∗∗ ​< ​0.01 (two-tailed).

a Missing data for n ​= ​1 PSD participant in the training group and n ​= ​1 PSD participant in TAU group.

b missing data for n ​= ​1 PSD participant in the training group and n ​= ​1 PSD participant in TAU group.

### 3.4 Effect of VR training on cognitive functioning

**Fig. 3:**
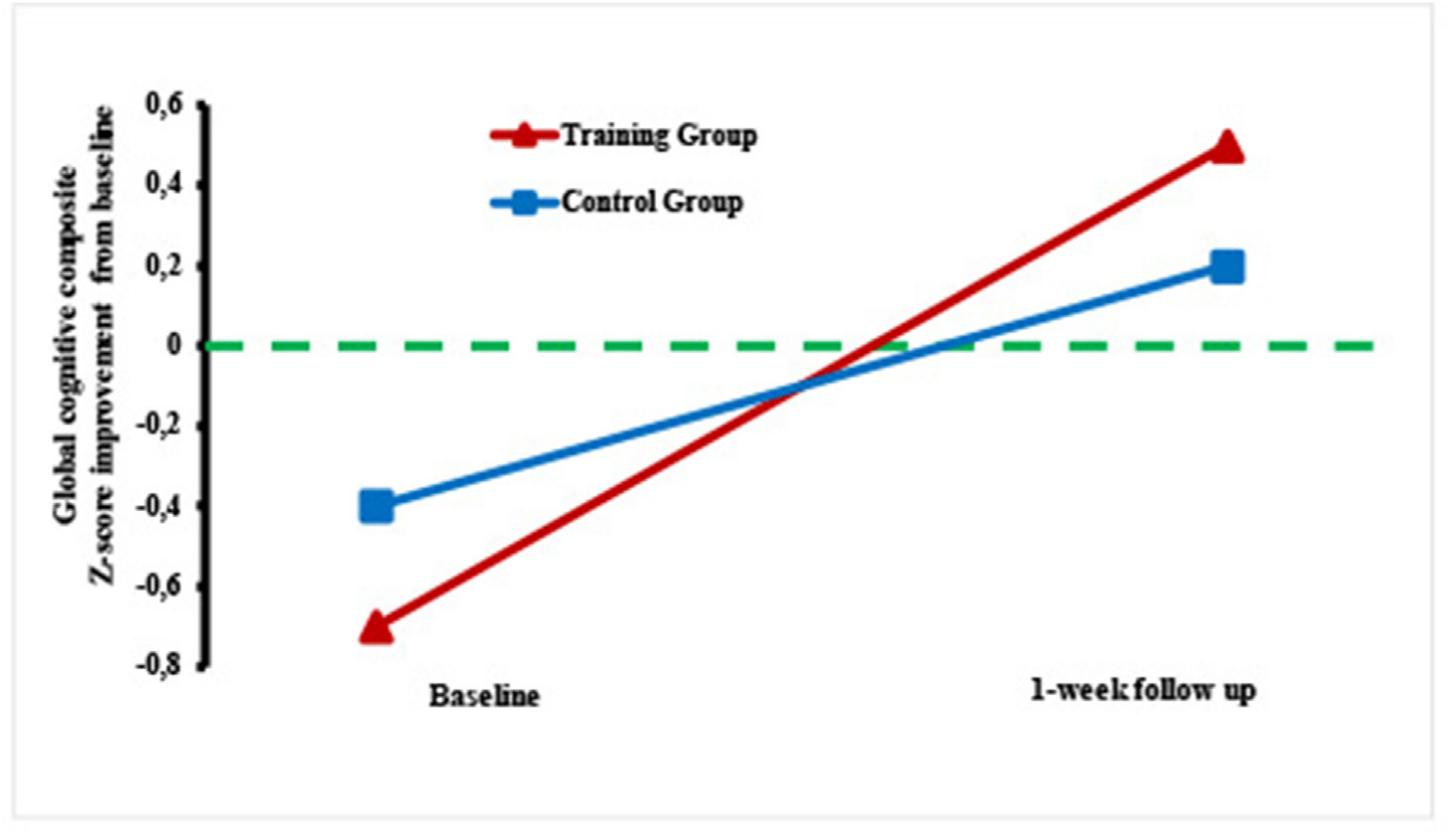
Improvement in the VR training group vs. TAU group on the CAVIR *test* cognitive composite score. Note. Improvement of the VR training group (mood disorders n ​= ​10; psychosis spectrum disorders n ​= ​11) vs. control group (MD n ​= ​10; PD n ​= ​11) on a CAVIR cognitive composite score (primary outcome). The Y-axis denotes average z-scores based on the mean and standard deviation of a healthy control group (HC) (n ​= ​40). HC M ​= ​0 and SD ​= ​1.

The analysis revealed a significant effect of one-week of VR-assisted cognitive training vs. TAU on the CAVIR global composite score with a large effect size (*F*(1, 38) ​= ​11,29 *p* ​= ​.002, *g* ​= ​1.04; Table 3 and Fig. 3). This effect prevailed after adjusting for subsyndromal depressive symptoms and negative symptoms (*p* ​= ​.02).

**Fig. 4:**
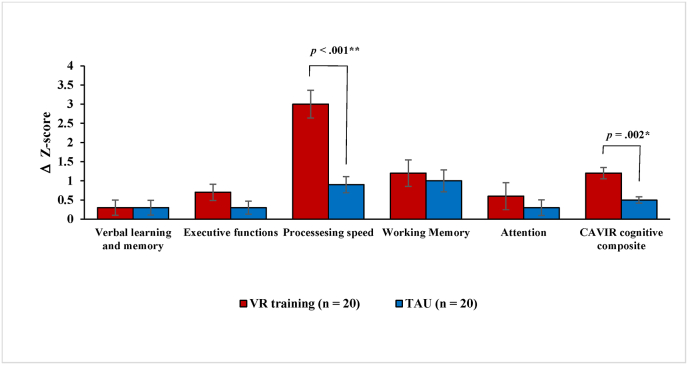
Improvement in the VR training group vs. TAU group on the global cognitive composite score and the five subdomains on the CAVIR test. Note. Improvement in the VR training group vs. TAU group on the five CAVIR subdomains and the global CAVIR composite score. The Y-axis denotes the average delta Z-score (Δ ​= ​pre-VR training minus post-VR training). The average Z-scores are based on the mean and standard deviation (SD) of a healthy control group (HC) (n ​= ​40; Miskowiak et al. in prep). The CAVIR cognitive composite score is derived by averaging the five z-transformed subtask scores. HC M ​= ​0 and SD ​= ​1. Error bars represent standard error of the mean. ∗p ​< ​.05; ∗∗p ​< ​.01.

For the individual CAVIR subdomains, VR-assisted training vs. TAU improved processing speed with a large effect size (*F*(1, 38) ​= ​22.78, *p* ​< ​.001, *g* ​= ​1.54; Table 3 and Fig. 4). This effect remained after adjusting for subsyndromal depressive symptoms and negative symptoms for MD and PSD patients, respectively (*p* ​= ​.01). There was no significant effect of treatment in the remaining CAVIR subdomains (*p*_*s*_ ≥. 21; Table 3 and Fig. 4).

For the neuropsychological domains, analyses showed no significant effect of VR-assisted training vs. TAU (*p*_*s*_ ​≥ ​.08; Table 3). However, there was a non-significant trend towards a treatment-related effect on verbal learning and memory (*p* ​= ​.09; Table 3). In contrast, there was a non-significant trend towards more improvement in the TAU group vs. the VR training group in processing speed (*p* ​= ​.08; Table 3).

Post-hoc correlation analysis revealed that lower global cognition, as reflected by a lower CAVIR composite score, at baseline was associated with a greater global cognitive improvement on the CAVIR test in the intervention group (*r* (20) ​= ​−0.7, *p* ​= ​.001).

## 4 Discussion

This proof-of-concept study investigated for the first time the feasibility and possible cognitive benefits of a novel fully immersive VR *prototype* kitchen training scenario in a sample of symptomatically stable patients with MD or PSD. In line with our first hypothesis, short-term VR-assisted cognitive training revealed good feasibility, as reflected by 100% of patients completing the training sessions, 75% completing all homework assignments and ratings of the VR training scenario as engaging, user-friendly, relevant and with minimal simulation sickens. In line with the second hypothesis, one week of VR-assisted cognitive training improved global cognition measured with the CAVIR test; a VR test of daily-life cognitive functions involving a different VR kitchen scenario. This effect on global cognition was driven primarily by improved accuracy on the CAVIR processing speed test. In contrast, no effects were observed on other VR subtests or on the traditional neuropsychological domains, except for a trend towards treatment-related improvement of verbal learning and memory.

The high completion rate and positive feedback regarding user-experience is in accordance with findings from our recent systematic review suggesting that cognitive programs using fully immersive VR generally have low attrition rates and high feasibility (Jahn et al., 2021). This is particularly promising considering how many CR studies suffer pronounced attrition rates (as high as 50%) and difficulties motivating participants to complete their assigned training (Lewandowski et al., 2017; Glenthoj et al., 2017; Bowie, 2019). Indeed, recent CR studies report a high dropout rate as an important limitation, suggesting that it may reflect low motivation and interventions not being well tolerated by participants (Lewandowski et al., 2017; Glenthoj et al., 2020; Ott et al., 2021). In a CR study by Piskulic et al. (2015) in young people at high risk of psychosis, the authors highlight loss of interest in the training as one of the main reasons for patients' treatment discontinuation. Similarly, CR studies in MD reported discontent with the training itself (e.g., training being either too boring or too burdensome) as one of the primary reasons for non-completion (Lewandowski et al., 2017; Ott et al., 2021). Together, these observations emphasize the critical need to explore more appealing and engaging CR methods such as VR, particularly when targeting younger patients who are typically already quite skilled in navigating computer platforms (Piskulic et al., 2015; Glenthoj et al., 2020). In the current study, about half of the participants in the training group noted that the VR scenario was fun and entertaining, highlighting how the training felt life-like and engaged all their senses at once. Participants also reported experiencing a moderate to high degree of ‘presence’ in the virtual kitchen scenario; an experience that is linked to increased engagement in VR which in turn is associated with higher perceived learning (Makransky and Lilleholt, 2018). Taken together, our findings are in accordance with literature suggesting that fully immersive VR has a significant potential for use in learning applications as it may be superior to other non-immersive platforms (e.g., computer training) in arousing, engaging and motivating learners (Makransky and Lilleholt, 2018; Makransky et al., 2019, 2020).

The finding of a significant treatment-related improvement on cognitive performance driven primarily by improved accuracy in the CAVIR processing speed test, suggests that short-term VR-assisted cognition training has the potential to produce some cognitive benefits. However, as the current study had no *active* control group with similar exposure to VR, we cannot not exclude the possibility that the improved performance on the CAVIR test in the active training group could be related to their longer VR exposure (albeit in a different VR training scenario) rather than cognitive improvement per se. Nevertheless, we find this unlikely because the CAVIR test and training scenarios differ substantially with regards to kitchen setup, stimuli, and subtask designs. Therefore, although both scenarios contain similar *types* of tasks, completing the training scenario does not give participants any direct advantages for completing the CAVIR test except when participants actively use the trained CR *strategies*. Hence, the improved CAVIR test performance in the training group likely reflects how participants actively applied learned CR strategies at follow up. However, it is likely that this strategy application was more easily applied in the ecologically valid CAVIR subtests than in the more abstract neuropsychological counterparts, which could explain why the treatment-related effect on the CAVIR processing speed test was not replicated on the neuropsychological domain of processing speed. The CAVIR processing speed test was modelled partially after a coding task like the SDMT, which can be considered a “dirty test” that includes both elements of working memory and processing speed (Sandry et al., 2021; McIntyre et al., 2014). Notably, much of the VR training focused on slowing down and being more mindful in the interest of higher accuracy during task performance. The improved accuracy in the CAVIR processing speed test therefore likely reflects greater use of strategies to aid associations between symbols and ingredients, i.e., an improvement of the working memory component of the subtest. However, this same strategy of slowing down in the interest of optimal accuracy may have been maladaptive on the neuropsychological processing speed tasks that to a greater extent favor speed (e.g., TMT-A). Indeed, correlation analyses revealed a mild correlation between the CAVIR processing speed test and the neuropsychological counterparts across the entire sample at baseline, which disappeared at follow up (see Supplementary materials Tables A.2 and **A.3** in Appendix A) suggesting that task performance diverged between the two groups at follow up, possibly due to the training group actively employing the practiced strategies. Taken together, the treatment-related improvement on the CAVIR test is thus more likely to reflect a therapeutic effect of training cognitive strategies rather than simple perceptual habituation to being in a VR kitchen scenario, which is consistent with literature showing that strategy-based learning may be a particularly effective aspect of VR based cognitive training (Jahn et al., 2021; Vass et al., 2020). Nevertheless, we cannot completely exclude a habituation to VR on a broader level. In our planned larger-scale randomized controlled trial (RCT) based on the current proof-of-concept findings, we will therefore include an *active* control group that is also exposed to the same VR environments but without training elements.

The lack of treatment-related improvement on the traditional neuropsychological test battery after one week of VR-based training was not unexpected. Indeed, the VR training program focused on very specific strategic skills related to daily life challenges, which may not be directly captured in more abstract neuropsychological tests. This is consistent with literature showing a generally poor correlation between neuropsychological tests and daily-life functioning (Van der Elst et al., 2008). Interestingly, we found in a recent systematic review, that greatest cognitive benefits of VR-based cognitive rehabilitation occurred when the interventions included more naturalistic settings involving task that resemble daily life activities (e.g., cooking a meal or shopping) (Jahn et al., 2021). In keeping with this, most participants in our training group reported finding the VR training useful outside the VR environment (e.g., when grocery shopping and cooking a meal), which could suggest a potential transfer of the acquired cognitive skills to daily life. This is notable because transfer is essential to aid patients’ application of trained skills to tackle cognitive challenges in daily life (Tsapekos et al., 2020; Miskowiak et al., 2022a) and there is a general lack of such transfer for most CR interventions (Woolf et al., 2022; Tsapekos et al., 2020; Miskowiak et al., 2022a). In our upcoming larger-scale RCT study, we will therefore include assessment of the possible functional implications of VR-related cognitive improvement as a secondary trial outcome. Interestingly, the severity of pre-treatment impairment in global cognition measured with the CAVIR test was associated with more cognitive improvement in the intervention group. This in line with previous findings that baseline cognition predicts response to pro-cognitive interventions (Miskowiak et al., 2017). Based on this finding and the recommendations by the ISBD Targeting Cognition Task Force (Miskowiak et al., 2017), we will therefore also pre-screen participants for cognitive impairment in our planned RCT to ensure an enriched sample.

A limitation of the study was that we used the same (rather than parallel) version(s) of the CAVIR test at baseline and follow-up, which induced a moderate learning effect with repeated testing. However, the test was the same for the active treatment and TAU groups, and it is therefore noteworthy that the active group showed an improvement that was *greater than* the improvement in the TAU group. Nevertheless, to minimize learning effects with repeated testing in future intervention studies, we have designed and will validate an alternate parallel CAVIR test with different test stimuli. Finally, it should be noted that both therapists and participants were unblinded in terms of allocation, which may have affected self-report measures due to expectation effects and introduced an experimenter bias. However, the assessment of cognitive performance in the CAVIR test is fully automated, rendering such bias unlikely.

In conclusion, we found that a novel fully immersive VR-based cognitive training platform is feasible and has potential to improve some aspects of cognition even with short-term training in patients with MD or PSD. Building on these proof-of-concept findings, we now plan to start a larger-scale RCT to investigate the efficacy of a four-weeks VR-assisted cognitive training programme in these patient groups. The perspective is a highly engaging cognitive training platform in VR that can aid bridging between CR interventions and daily life cognitive challenges for patients with MD or PSD to improve their functioning and quality of life.

## Author contributions

All authors met all four ICMJE criteria for authorship. Andreas Jespersen and Kamilla Miskowiak were involved in the initial conception and design of the study, the development of the VR training platform and analyzing the data. Andreas Jespersen wrote the first draft of the article in collaboration with Kamilla Miskowiak. Anders Lumbye was responsible for the technical development of the VR training platform with the input from Kamilla Miskowiak and Andreas Jespersen. Andreas Jespersen, Isabella Røen, Louise Glenthøj and Merete Nordentoft contributed to the acquisition and interpretation of the data. All authors read and approved of the final version of the manuscript and agreed to be accountable for all aspects of the work.

## Declaration of competing interest

The authors declare the following financial interests/personal relationships which may be considered as potential competing interests: Kamilla Miskowiak has received consultancy fees from Lundbeck, Angelini and Janssen-Cilag in the past three years. Andreas Jespersen, Louise Glenthøj, Merete Nordentoft, Isabella Røen and Anders Lumbye report no conflicts of interest.
