# Supplementary material for: Feasibility and effect of an immersive virtual reality-based platform for cognitive training in real-life scenarios in patients with mood - or psychotic disorders: A randomized, controlled proof-of-concept study
Source: Neurosci Appl. 2023 Feb 4;2:101120. doi: 10.1016/j.nsa.2023.101120 (PMC12244015; doi:10.1016/j.nsa.2023.101120)
Supplement: Multimedia component 1 [file mmc1.docx]

**Appendix A: Supplementary material**

*Supplementary methods: Subtasks in the Cognition Assessment in Virtual Reality (CAVIR) test*

Verbal learning is assessed in task 1, in which participants are shown a list with ingredients and instructed to memorize these and take them out of the fridge. Performance is measured by the number of correct ingredients remembered (score range: 0-10). Executive functions are assessed in task 2, in which participants are required to plan and select the order in which to perform different sub-tasks involved in cooking a meal to finish before their guests’ arrival. Here, performance is measured the number of correctly placed tasks that ensure timely completion of the tasks (score range: 0-7). Processing speed is assessed in task 3, in which participants place as many correct ingredients as possible in a pot within 90 seconds based on a key of symbols matching every ingredient (no score range). Working memory is assessed in task 4, in which participants observe and memorize the location of cutlery and flatware in the kitchen cupboards and drawers. Performance is measured by the number of drawers opened until all cutlery and flatware is found (lower indicating better performance). Finally, sustained attention is measured in task 5, in which participants are required to repeatedly check the lasagne in the oven in response to a specific combination of visual and auditive cues while ignoring irrelevant stimuli. Performance is measured by the number of correct responses (score range: 0-20).

*Supplementary methods: statistics*

Outlying z-scores (> 4 SDs below HC mean) were truncated to z =-4.0 to limit the impact of extreme scores while still allowing variability in the data in line with previous studies from our group (9). For tests, in which lower scores indicated better performance (i.e., latency in the CAVIR task 2; TMT tests, RVP, SWM and OTS), the scores were inversed before standardization to ensure that that all scales had the same direction. For the CAVIR, five cognitive domains were calculated by averaging the z-transformed scores within each of the five sub-tasks (see table 1 for an overview of the CAVIR cognitive domains). A global CAVIR composite score was then calculated by averaging the five domains. Data was excluded for participants who had misunderstood individual CAVIR tasks, as assessed by a performance >3 SD below mean of their respective group. For participants with missing CAVIR data, the global CAVIR composite was calculated by averaging the remaining domains. Five cognitive domains were also calculated based on the z-transformed neuropsychological test scores within the respective domains (see Table 1). A global neuropsychological composite score was calculated by averaging these five cognitive domains.

**Table A.1**. Overview of the Cognition Assessment in Virtual Reality (CAVIR) tests and associated standardised neuropsychological tests assessing processing speed, attention, verbal learning and memory, working memory and executive functions. From Miskowiak et al. 2022.

| **Cognitive domains** | **CAVIR sub-task measures** | **Neuropsychological tests** |
| --- | --- | --- |
| Processing speed | Number of correct ingredients placed in the stove pot within 90 seconds. | RBANS coding test Trail Making A |
| Verbal Learning and memory | Number of correctly remembered ingredients chosen in the fridge. Number of false positives (wrong ingredients chosen; score inversed) | RAVLT subtests (IV total, Immediate recall, delayed recall, recognition) |
| Working memory | Number of drawers opened until all cutlery and flatware has been found (score inversed) Latency when solving the task (score inversed) | WAIS Letter-number sequencing  Spatial working memory error (CANTAB)  Spatial working memory strategy (CANTAB) |
| Executive function | Number of cooking tasks correctly placed on a to-do list.  Latency when solving the task (score inversed) | Trail Making B One-touch stockings of Cambridge mean choices to correct (CANTAB)  Fluency test (S and D) |
| Attention | Correct hits (opening the stove door when the light on the stove turns green combined with the correct sound)  Number of false positives (opening the stove door when seeing/hearing foil stimuli; score inversed) | Rapid visual processing accuracy (CANTAB) Rapid visual processing latency (CANTAB) RBANS digit span |

*Supplementary results:*

Key insights derived from the 13 out of 20 participants in the training group who chose to elaborate on their experience on the written feedback form.

- *It is fun with VR. I learn better when it is fun. The tasks in VR felt more like a game and that helps you relax more.* **24 y/o participant with schizophrenia**
- “*It was much easier to apply the strategies today (second training session red.). It is good with repetition. VR is fun and very realistic.* *It seems like you use more parts of the brain when training with the headset because more senses are involved. It could be fun to do the tasks with the headset at home. It feels like the training is working and it is fun*.” **50 y/o participant with previous depression**
- *“The strategy with visualizing a funny story in VR helped me in the supermarket. I often forget to write a shopping list and have to remember what to buy”.* **33 y/o participant with previous depression.**
- *The strategies helped when I when shopping and had to find my way around in Rødovre mall and find my way back to the bus stop*. **22 y/o participant with schizophrenia**
- *I tried using the strategy with slowing down when I had to learn a new language and when I went shopping. It was fun to train in virtual reality and a lot like playing a game. I would have liked to train in other scenarios than only a kitchen though, because I do not experience many difficulties here*. **25 y/o participant with schizotypal disorder**
- *It was nice that during the virtual reality training, I did not focus on the world around me which can be very stressful. For instance, I can think a lot about what other people are thinking. In VR I shut everything out and concentrated on the task I had to solve. I felt like I was more present [compared to computer-based exercises Red.] like in the real world. I could imagine a great potential with VR for training and trying out stuff, practice what is difficult in daily life such as taking the bus, shopping, working etc.* **38 y/o participants with bipolar disorder**
- *I used the strategy with thinking in pictures and categorizing when I had to organize some papers and clean up. Also, when I had to remember what to pack and buy in the supermarket. Slowing down also helped me when I listen to others.* **45 y/o participant with bipolar disorder.**
- *I helped me to use the strategy of slowing down at my work. I also tried to visualize items when I went shopping. I have not made a dish at home both times because we went out to eat a couple of evenings.* **55 y/o participant with bipolar disorder**
- *I was very excited about the VR headset. It was fun and it helped me that it felt so realistic. The scenario with the kitchen was a very good exercise for me as I have a lot of difficulties remembering where things are located. The strategy with visualizing a pattern and finding similarities helped me a lot*. **32 y/o participant with bipolar disorder**
- *The strategy with the weird story has been pretty helpful for getting information such as ingredients to stick better in my memory, so that is a strategy I will try to use in other situations. But the other strategies did not help me much for the things that I often forget every day such as calling people and appointments*. **26 y/o participant with schizotypal disorder**
- *I think it is fun to train in VR. It is important that it is motivating, especially if you have to train at home. It is important that you feel some kind of reward. This is very important because otherwise you do not want to do the training*. **24 y/o participant with bipolar disorder.**
- *I used the strategy with visualizing a pattern when I had to remember where I had parked my car. I have also experienced that I remember things better if I make a story in my head. I think it [the training Red.] was pretty fun and helpful and I liked the strategies***. 21 y/o participant with bipolar disorder.**
- *When I went shopping, I used the story, which helped a lot with remembering the ingredients. I am very visual in the way I remember things, so it is a funny method. I think it was fun to use VR to train my memory. However, in the VR kitchen, it was quite short time you had to make a story. But all in all, I think it was fun and useful*. **31 y/o participant with schizophrenia**

**Figure A.1:** Baseline performance of the VR training group vs. TAU group on the global cognitive composite score and the five subdomains on the CAVIR test.

*Note. Baseline performance on the VR training group vs. TAU group on the five CAVIR subdomains and the global CAVIR composite score. The Y-axis denotes the mean cognition z-score for the patient group based on the mean and standard deviation (SD) of the healthy controls (HC; n=40). The CAVIR cognitive composite score is derived by averaging the five z-transformed sub-task scores. Bars represent mean composite scores for patients relative to HC. HC M = 0 and SD =1 *p<0.05; ** p<0.01.*

| **Table A.2**. Correlations *at baseline* between Cognition Assessement in Virtual Reality (CAVIR) test performance and traditional tests across the entire sample | | | | | | |  |
| --- | --- | --- | --- | --- | --- | --- | --- |
|  | **Traditional neuropsychological domains** | | | | | | |
| **CAVIR test cognitive domain** | Verbal learning/ memory | Executive functions | Processing speed | | Working memory | Attention | Global cognition |
| Verbal learning / memory | **.36*** | .28 | | .16 | **.46**** | .09 | **.38**** |
| Executive functions | .13 | .08 | | .04 | .04 | .09 | .05 |
| Processing speed | **.35*** | .24 | | **.39*** | .30 | .21 | **.43**** |
| Working memory | **.33*** | .16 | | .27 | **.43**** | .14 | **.34*** |
| Attention | .18 | .16 | | .17 | .11 | .15 | .26 |
| CAVIR global cognition | **.45**** | .30 | | .31 | **.42**** | .04 | **.46**** |
|  |  |  | |  |  |  |  |
|  |  |  | |  |  |  |  |
|  |  |  | |  |  |  |  |
| **Table A.3.** Correlations *at follow up* between Cognition Assessement in Virtual Reality (CAVIR) test performance and traditional tests across the entire sample | | | | | | |  |
|  | **Traditional neuropsychological domains** | | | | | | |
| **CAVIR test cognitive domain** | Verbal learning/ memory | Executive functions | | Processing speed | Working memory | Attention | Global cognition |
| Verbal learning / memory | **.39*** | **.39**** | | **.51**** | **.49**** | .31 | **.56**** |
| Executive functions | **.49**** | .02 | | .01 | .18 | .02 | .11 |
| Processing speed | **.42*** | .17 | | .21 | .20 | .25 | .31 |
| Working memory | **.40*** | .16 | | **.45**** | .20 | .12 | **.38*** |
| Attention | .05 | .29 | | .26 | .24 | .09 | .25 |
| CAVIR global cognition | **.48**** | .27 | | **.43**** | .31 | .28 | **.48**** |
